# Effect of time and titer in convalescent plasma therapy for COVID-19

**DOI:** 10.1016/j.isci.2021.102898

**Published:** 2021-07-22

**Authors:** Paola de Candia, Francesco Prattichizzo, Silvia Garavelli, Rosalba La Grotta, Annunziata De Rosa, Agostina Pontarelli, Roberto Parrella, Antonio Ceriello, Giuseppe Matarese

**Affiliations:** 1 IRCCS MultiMedica, 20138 Milano, Italy; 2 Istituto per l'Endocrinologia e l'Oncologia Sperimentale, Consiglio Nazionale delle Ricerche (IEOS-CNR), 80131 Naples, Italy; 3 Dipartimento di Malattie Infettive ed Emergenze Infettive, Divisione di Malattie Infettive Respiratorie, Ospedale Cotugno, AORN dei Colli, 80131 Naples, Italy; 4 Treg Cell Lab, Dipartimento di Medicina Molecolare e Biotecnologie Mediche, Università di Napoli Federico II, 80131 Naples, Italy

**Keywords:** systems medicine, virology

## Abstract

The clinical benefit of convalescent plasma (CP) for patients with coronavirus disease (COVID)-19 is still debated. In this systematic review and meta-analysis, we selected 10 randomized clinical trials (RCTs) and 15 non-randomized studies (total number of patients = 22,591) of CP treatment and evaluated two different scenarios: (1) disease stage of plasma recipients and (2) donated plasma antibody titer, considering all-cause mortality at the latest follow-up. Our results show that, when provided at early stages of the disease, CP significantly reduced mortality: risk ratio (RR) 0.72 (0.68, 0.77), p < 0.00001, while provided in severe or critical conditions, it did not (RR: 0.94 [0.86, 1.04], p = 0.22). On the other hand, the benefit on mortality was not increased by using plasma with a high-antibody titer compared with unselected plasma. This meta-analysis might promote CP usage in patients with early-stage COVID-19 in further RCTs to maximize its benefit in decreasing mortality, especially in less affluent countries.

## Highlights

• The benefit of convalescent plasma (CP) for patients with COVID-19 is still debated
• Only when provided at early disease stages, CP reduced COVID-19 mortality
• CP benefit on mortality does not increase when selecting high-antibody titers
• Early treatment with CP may maximize its clinical benefit

## Graphical abstract

**Figure:**
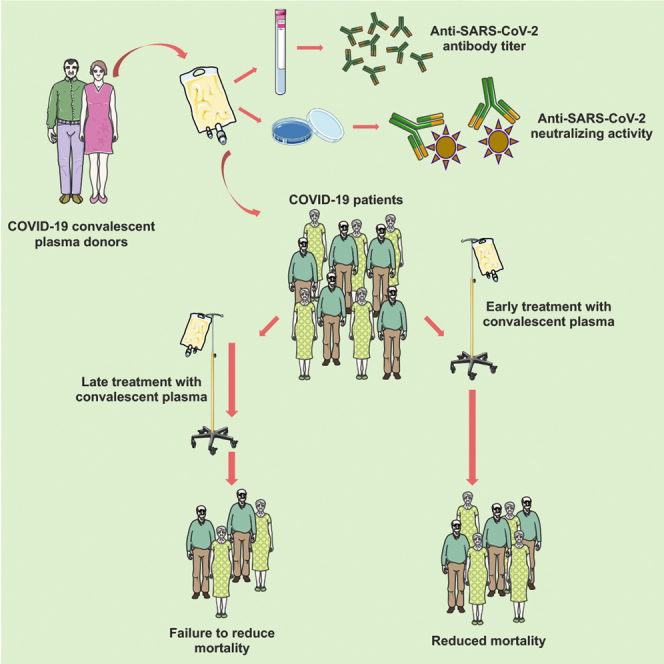

## Introduction

Coronavirus disease (COVID)-19 has affected more than 180,000,000 subjects and caused almost 4,000,000 deaths, as of July 1^st^, 2021, according to the Coronavirus Resource Center at Johns Hopkins University. The disease, caused by SARS-CoV-2, is usually accompanied by the development of mild, flu-like symptoms; however, a minor portion of patients, usually the elderlies and those with pre-existing comorbidities (i.e. diabetes, obesity, and hypertension), develop life-threating complications, such as severe pneumonia, cardiovascular diseases, and multi-organ failure which eventually lead to death (Lu et al., 2020; Zhou et al., 2020a, 2020b; Zhu et al., 2020). Vaccination is considered the most effective strategy to protect the frail population but its prophylactic approach finds no use in the therapeutic setting, where the identification of effective antivirals and other therapies able to limit the deleterious consequences of COVID-19 complications is crucial (Davis et al., 2020). In circumstances in which the virus induces an immune response entailing the production of neutralizing antibodies, the collection of plasma from a convalescent donor and its passive transfusion to another patient (an approach known since the beginning of 1900) (Marson et al., 2020) has proven to be a powerful and feasible therapeutic strategy for the clearance of viremia (Mair-Jenkins et al., 2015). The use of plasma from convalescent patients with COVID-19 was indeed proposed from the very beginning as a treatment to halt virus progression and promote favorable outcomes (Chen et al., 2020) and authorized by the Food and Drug Administration (FDA) as an investigational drug for contrasting the novel pathogen (https://www.fda.gov/vaccines-blood-biologics/investigational-new-drug-ind-or-device-exemption-ide-process-cber/recommendations-investigational-covid-19-convalescent-plasma).

However, randomized clinical trials (RCTs) and cohort studies have provided with conflicting results and three meta-analysis found a consistent benefit (Klassen et al., 2020), a low-quality evidence for mortality reduction (Sarkar et al., 2021), and no substantial benefit on a range of possible outcomes (Chai et al., 2020), respectively. A number of reasons have been proposed to explain the observed differences in the outcomes of patients with COVID-19 treated with convalescent plasma (CP). In particular, it has been hypothesized that time from hospital admission to CP treatment (the earlier in the course of disease, the better) and/or prior selection of plasma with high-antibody titer (or *in vitro*-tested SARS-CoV-2 neutralization activity) might be key determinants of CP efficacy (Joyner et al., 2021; Tobian and Shaz, 2020). Since none of the previous meta-analyses has directly tested this hypothesis, we have here conducted a pragmatic, rationale-based systematic review and meta-analysis to examine the effect of CP in patients with COVID-19 by considering two different scenarios: (1) the disease stage of plasma recipients and (2) the donated plasma antibody titer; we have then extrapolated one outcome, *i.e.* all-cause mortality at the longest possible follow-up.

## Results

The inclusion flow is presented in Figure S1. Upon identification of 753 univocal records, we selected 40 pertinent manuscripts. During the screening phase of these manuscripts, 15 were excluded: three studies did not report mortality data or compared mortality rate with national registries (Bradfute et al., 2020; Dulipsingh et al., 2020; Perotti et al., 2020); for two studies, we were unable to assess our inclusion criteria (Anakli et al., 2021; Skrip et al., 2020); seven had no comparative group (Dulipsingh et al., 2020; González et al., 2020; Jaiswal et al., 2021; Madariaga et al., 2020; Olivares-Gazca et al., 2020; Tremblay et al., 2020; Valentini et al., 2020); and three studies made use of plasma from non-convalescent, generic donors (DeSimone et al., 2021; Faqihi et al., 2020; Kamran et al., 2021). Of the 25 studies included for the quantitative meta-analysis, 10 were RCTs (Agarwal et al., 2020; AlQahtani et al., 2020; Avendano-Sola et al., 2020; Balcells et al., 2021; Gharbharan et al., 2020; Horby et al., 2021; Li et al., 2020a; Libster et al., 2021; Rasheed et al., 2020; Simonovich et al., 2020), while the other 15 were non-randomized studies with different designs (Abolghasemi et al., 2020; Alsharidah et al., 2021; Altuntas et al., 2021; Budhiraja et al., 2021; Donato et al., 2021; Duan et al., 2020; Hegerova et al., 2020; Joyner et al., 2021; Liu et al., 2020; Omrani et al., 2021; Salazar et al., 2020a; Shenoy et al., 2021; Xia et al., 2020; Yoon et al., 2021; Zeng et al., 2020), as detailed in Table S1. Two studies did not have a control group; nonetheless, one provided the relative mortality data upon multiple comparisons between patients receiving high *vs*. low titer plasma and between early (≤3 days from hospitalization) and late treatment (Joyner et al., 2021), or comparing the effects of immediate plasma treatment toward plasma treatment only upon patient deterioration (deferred treatment) (Balcells et al., 2021), and they were thus both included. Similarly, Salazar et al. provided multiple comparisons considering treatment time and plasma titer, all compared with a matched control group without treatment (Salazar et al., 2020a) and other three studies contained subgroup data for comparing early and late treatment, based on the severity of enrolled patients (Alsharidah et al., 2021; Donato et al., 2021; Horby et al., 2021). Appropriate mortality end points were thus extracted from these studies according to the different scenarios. All the other studies did not provide data from similar subgroups. Thus, they were categorized as (1) late *vs*. early treatment; and (2) use of high-titer plasma *vs*. studies only checking for the presence/absence of antibodies without further characterization. To make the first classification, priority was given to disease severity of the enrolled population. If patients were not on any form of mechanical ventilation and did not need supplemental oxygen, the treatment was considered “early”. In case of exclusive enrollment of intensive care unit or ventilated patients, the treatment was instead considered “late”. In case of a heterogeneous population, the discriminating parameter was time to treatment, measured as median days from hospitalization to treatment. Given the indications provided in one study (Joyner et al., 2021), we applied the criteria of ≤3 days to categorize the treatment as early. As a result, we obtained 11 studies as early and 16 as late (Table S1). We were unable to categorize one study (Gharbharan et al., 2020), since the enrolled population was heterogeneous and we found no clear indication of hospitalization to treatment time. For the second pre-specified scenario, 12 studies made use of high-titer plasma (independently of whether plasma was examined pre- or post-usage) albeit with different cutoff and methodologies, 6 studies only checked for the presence/absence of antibodies, and other 4 studies did not check (Table S1). As above, 2 studies provided internal comparisons considering treatment plasma titer (Joyner et al., 2021; Salazar et al., 2020a); instead, we decided not to categorize one multicenter study (Budhiraja et al., 2021), since it used either positive only or high-titer plasma, depending on the specific center and was thus considered heterogeneous. The assessment of bias risk inside the included studies is summarized in Table S2.

Overall, we collected data from 22,591 patients, with a 63.8% prevalence of male sex (gender information was not provided in one study (Hegerova et al., 2020)) and a weighted age average (by approximation of median to mean value in the studies for which only the former parameter was reported) of 62.3 years (in three studies age was not provided as mean for each group but divided into different strata and was not included in the overall mean age calculation) (Budhiraja et al., 2021; Joyner et al., 2021; Salazar et al., 2020a).

**Figure 1:**
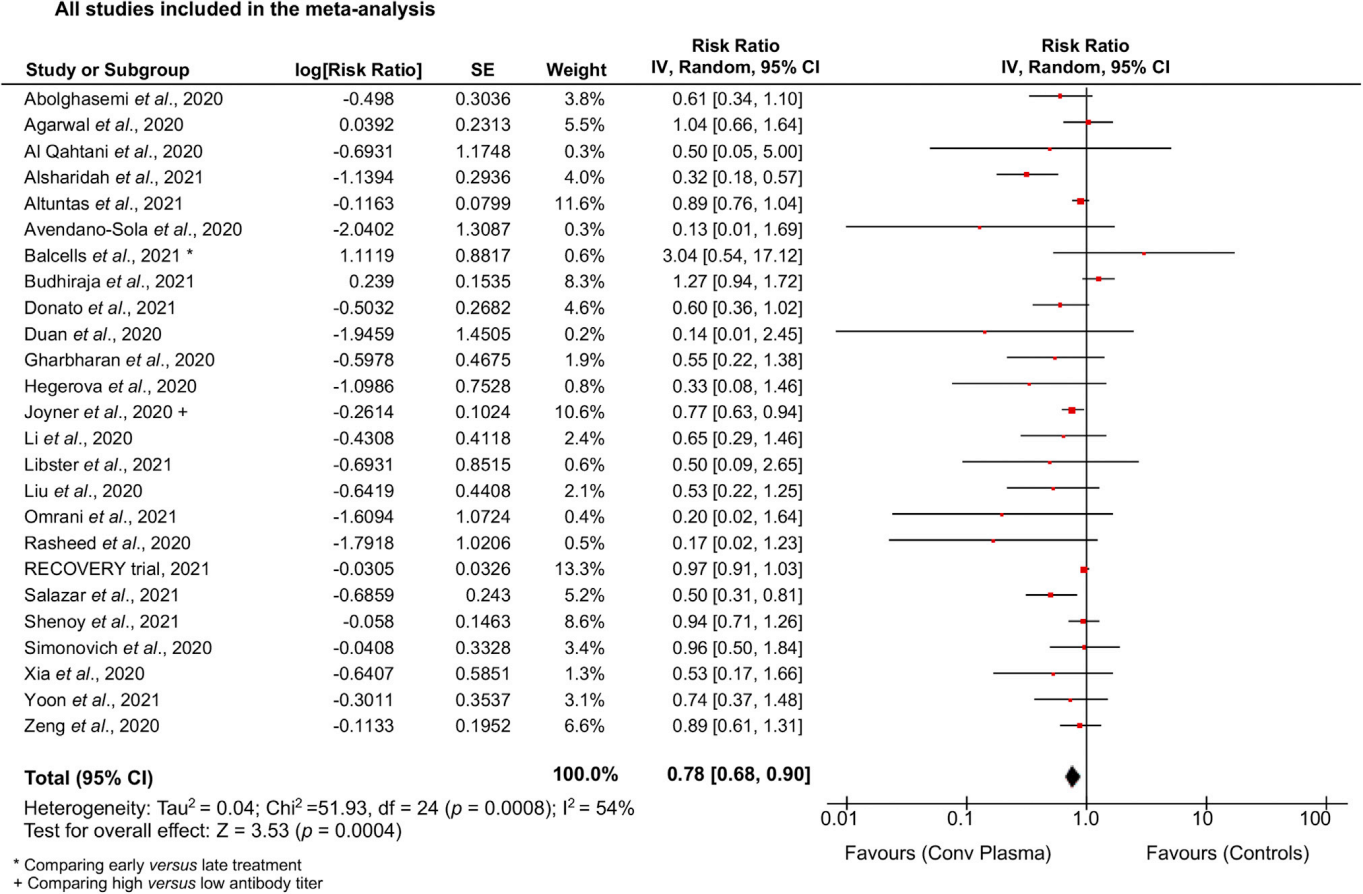
Benefit of convalescent plasma therapy for COVID-19 considering all studies Forest plot summarizing the effect of convalescent plasma vs standard of care or placebo or no treatment on mortality incidence in patients with COVID-19 considering all the available studies. ∗ For Balcells et al., data were from the comparison between early vs late treatment; + For Joyner et al., data were from the comparison between high and low antibody titer plasma.

When taking in account the totality of the studies, compared with no treatment, placebo or standard of care, CP was able to reduce mortality by a risk ratio (RR) of 0.78 (confidence interval [CI]: 0.68, 0.90, p for overall effect p = 0.0004) with significant heterogeneity across studies (I^2^ = 54%, p = 0.0008) (Figure 1). A funnel plot with all the included studies was used to evaluate the risk of publication bias (Figure S2); then a sensitivity analysis performed by excluding the studies with different design and those falling outside the 95% CI in the funnel plot demonstrated less evident effect with borderline statistical significance (RR: 0.94 [0.89, 1.00], p = 0.04; I^2^ = 26%, p = 0.13) (Figure S3). When considering only RCTs (Agarwal et al., 2020; AlQahtani et al., 2020; Avendano-Sola et al., 2020; Balcells et al., 2021; Gharbharan et al., 2020; Horby et al., 2021; Li et al., 2020a; Libster et al., 2021; Rasheed et al., 2020; Simonovich et al., 2020), CP failed to show any substantial effect on mortality (RR: 0.96 [0.91, 1.03], p = 0.24; I^2^ = 13%, p = 0.32) (Figure S4A).

**Figure 2:**
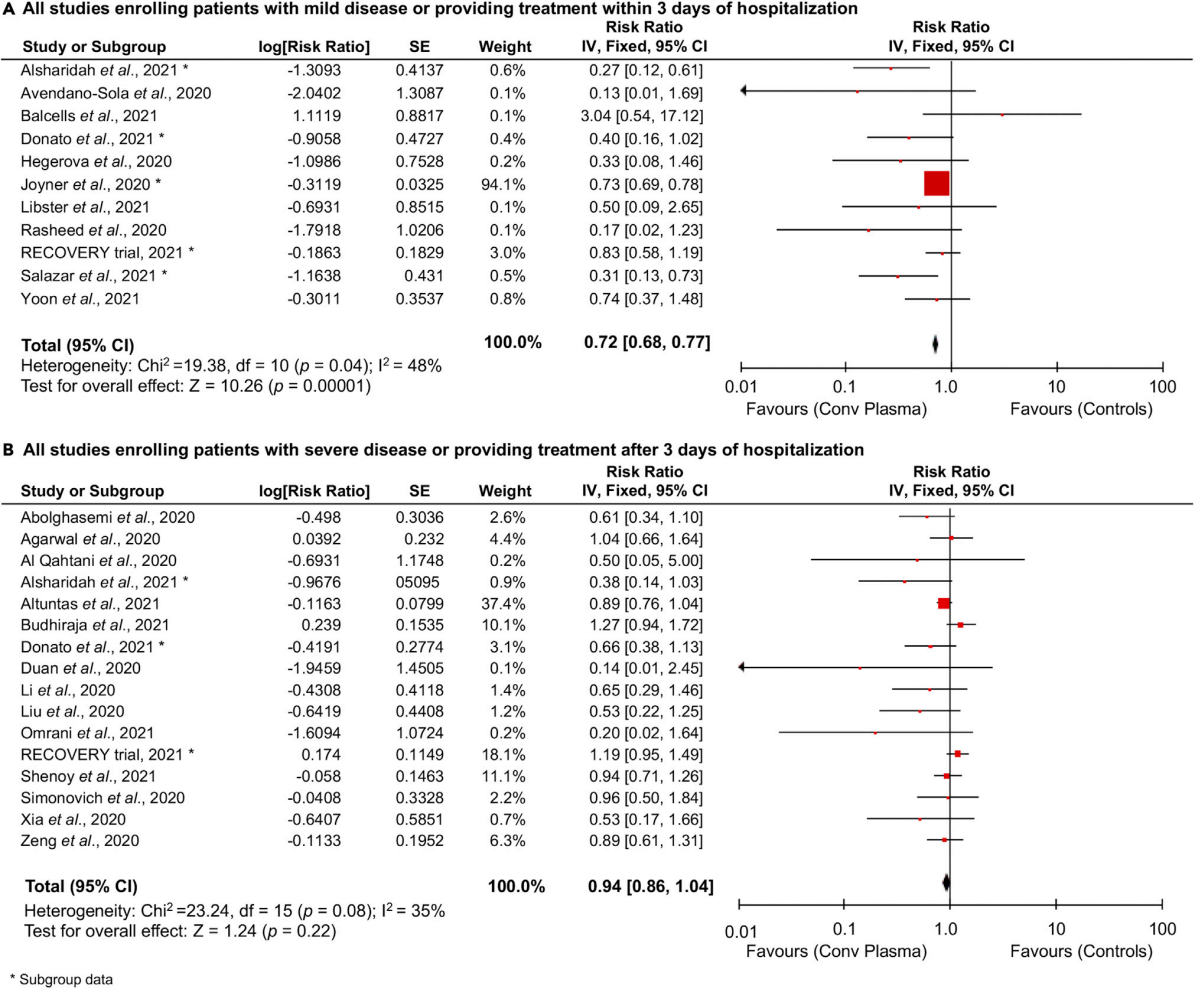
Effect of time in convalescent plasma therapy for COVID-19 Forest plots summarizing the effect of convalescent plasma vs standard of care or placebo or no treatment on mortality incidence in patients with COVID-19 considering all studies providing early treatment (A), or all the other studies providing plasma therapy at later stages (B). ∗ Subgroup data.

On the other hand, when provided at early stages of the disease or within 3 days of hospitalization, CP was able to significantly reduce mortality (RR: 0.72 [0.68, 0.77], p < 0.00001; I^2^ = 48%, p = 0.04) (Figure 2A), whereas provided at later stages of the disease (severe or critical conditions) or after 3 days of hospitalization, failed to show any efficacy in decreasing mortality (RR: 0.94 [0.86, 1.04], p = 0.22; I^2^ = 35%, p = 0.08) (Figure 2B). Similar results were obtained when RCTs were stratified based on time to treatment, with discrete, although not significant, reduction of mortality for studies providing treatment at early, but not late, stages of the disease (RR: 0.79 [0.57, 1.11], p = 0.17; I^2^ = 42%, p = 0.14) compared with (RR: 1.07 [0.89, 1.29], p = 0.47; I^2^ = 0%, p = 0.43) (Figures S4B and S4C).

**Figure 3:**
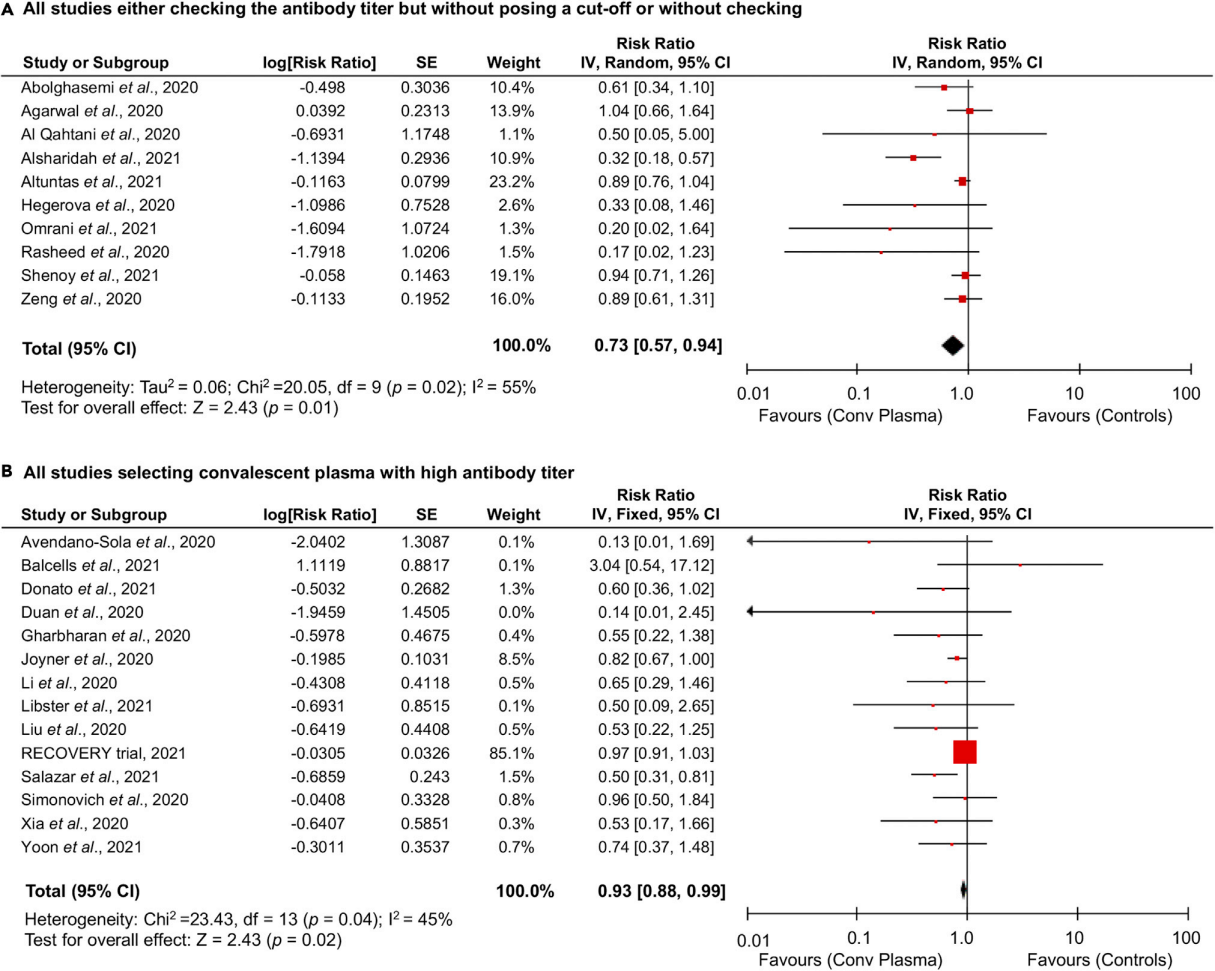
Effect of antibody titer in convalescent plasma therapy for COVID-19 Forest plots summarizing the effect of convalescent plasma vs standard of care or placebo or no treatment on mortality incidence in patients with COVID-19 considering all studies using plasma samples only screened for the presence of antibodies or with no check (A), and all the other studies using plasma samples selected for high-antibody titers (B).

In order to test the hypothesis that the use of high-titer plasma may provide with higher efficacy compared with plasma only screened for the presence of antibodies, we analyzed separately the studies with this divergent characteristic. When considering all studies, the treatment benefit on mortality did not differ for studies in which plasma was only screened for the presence of antibodies (or not checked) compared with studies in which a selection of plasma was based on a specific high-titer cutoff (RR: 0.73 [0.57, 0.94], p = 0.01; I^2^ = 55%, p = 0.02) *versus* (RR: 0.93 [0.88, 0.99], p = 0.02; I^2^ = 45%, p = 0.04) (Figures 3A and 3B). Similar conclusions were reached when we restricted the approach to RCTs and failed to observe a significant difference (RR: 0.93 [0.60, 1.43], p = 0.74; I^2^ = 40%, p = 0.19 for RCTs not posing a cut-off and RR: 0.80 [0.61, 1.04], p = 0.10; I^2^ = 0%, p = 0.45 for the other RCTs) (Figures S4D and S4E).

## Discussion

The ongoing SARS-CoV-2 pandemic is affecting millions of people around the globe and the health systems worldwide are struggling to tackle the emergence related to curbing the spread of this novel infectious agent and rapidly developing effective care strategies. As of today, few therapeutic options have demonstrated tangible benefit on hard outcomes (RECOVERY Collaborative Group et al., 2021). While CP therapy has been proposed from the very beginning as a potential tool to minimize the consequences of COVID-19, definitive results regarding its efficacy have not been yet provided (Chai et al., 2020). The emergency situation prompted expeditious study designing, with diversified primary outcomes and no careful scrutiny of the populations enrolled; as a consequence, the conflicting results might be ascribed to the marked heterogeneity of trials and observational reports.

First of all, the identification of the most appropriate treatment timing might have not been properly considered in all cases. Progression of COVID-19 is now believed to be characterized by different phases: the first one is denoted by acute viral replication, then, in case of ineffective viral clearance, a second phase can be marked by an aberrant immune response translating into a hyper-inflammatory reaction which is currently believed to be the main driver of worst outcomes; after that, the patient can either recover or go through organ dysfunction and death (Bonafe et al., 2020). Intuitively, an immune suppressive approach is suited for the second phase of COVID-19, while a therapy aimed at promoting viral clearance, such as that with CP, should be provided as early as possible during the course of the infection. All the more reason, since a consistent decline in viral load during the second phase of the disease has been reported in severe patients with COVID-19 (Cevik et al., 2021) and there are controversial findings about the actual presence of SARS-CoV-2 RNA during multi-organ failure that may instead be induced by aberrant levels of inflammatory mediators (the cytokine storm), endothelial dysfunction and coagulation abnormalities (Huang et al., 2020). On the other hand, an RCT, become public soon after the end-day of our meta-analysis, demonstrated that CP was able to significantly decrease the levels of interleukin-6, tumor necrosis factor-α, and interferon (IFN)-γ, calling for more studies on the immunomodulatory effect of this treatment on COVID-19-related cytokine storm (Pouladzadeh et al., 2021). Furthermore, a very recent retrospective study showed a survival benefit in the administration of CP also to COVID-19 patients with hematologic cancers (Thompson et al., 2021).

We have here analyzed two parameters associated with CP treatment: timing and antibody screening before clinical use. Through the first scenario (i.e. time to treatment, or the disease stage of plasma recipients), the present meta-analysis substantiates the clinical framework of early plasma transfusion for SARS-CoV-2 infected patients. The lack of significance observed when restricting this analysis only to RCTs might be ascribable to the low number of events reported in such trials. However, the observation that 4 of 5 RCTs had a non-significant trend toward a benefit for CP corroborates the design of further trials to assess this hypothesis. In addition, this result is consistent with the experiences accumulated with both SARS-CoV-1 infection and severe influenza that show how patients receiving CP transfusion early after symptom onset had better outcomes and revealed consistent evidence for a reduction in mortality (Cheng et al., 2005; Mair-Jenkins et al., 2015). Of note, the design of trials testing the effect of monoclonal antibodies in patients with COVID-19 is focusing exclusively, at present, on the effect of such therapies in patients with early stages of the disease (Cohen, 2021).

On the other hand, through the second considered scenario, *i.e.* donated plasma antibody titer, our study does not support the idea that plasma samples with higher titers of SARS-CoV-2 antibodies may be markedly more efficacious compared to unscreened plasma. It is important to underline here, though, that five (Duan et al., 2020; Li et al., 2020b; Liu et al., 2020; Simonovich et al., 2020; Yoon et al., 2021) of the 14 studies (36%) categorized as using high-antibody titer in Figure 3B, initiated plasma treatment at late stages of disease, possibly confounding the clean effect of plasma selection. Furthermore, although two studies evaluated here (one RCT (Simonovich et al., 2020) and one observational cohort study (Donato et al., 2021)) showed a significant correlation between the titer of total and that of *in vitro* neutralizing SARS-CoV-2 antibodies, the hypothesis that plasma containing higher titers of total antibodies may be more efficient in infection neutralization have not been yet exhaustively examined, hence the functional relevance of these observations remains questionable. Actually, viral neutralization and anti-spike protein antibodies in plasma donors were more recently found highly variable and poorly correlated with each other (Janaka et al., 2021). The possibility that patients suffering with worst clinical symptoms, reported to produce higher antibody titers (Benner et al., 2020; Boonyaratanakornkit et al., 2021; Bosnjak et al., 2020; Dogan et al., 2021; Klein et al., 2020; Mehew et al., 2020; Salazar et al., 2020b; Terpos et al., 2020; Wardhani et al., 2021), may be more suitable plasma donors, also needs to be fully assessed. The existing literature on clinical predictors of high-titer neutralizing antibodies strongly suggests that older age, male sex, and hospitalization are the main proxies to select plasma donor recruitment (Focosi and Franchini, 2021; Gontu et al., 2021). Additional hypotheses formulated to increase plasma efficacy rely also on the assumption that pooled plasma or other strategies to increase the variety of SARS-CoV-2 antigens targeted by transfused antibodies increase the effectiveness of plasma therapy (Focosi et al., 2020). Furthermore, since spike-specific and neutralizing antibodies were dramatically higher following a single vaccination after COVID-19 infection, compared to values seen with COVID-19 infection alone, recovered COVID-19 subjects who are vaccinated may make ideal candidates for CP donation (Vickers et al., 2021).

Among the studies evaluated here, only 3 manuscripts reported the use of plasma pooled from multiple donors (Agarwal et al., 2020; Simonovich et al., 2020; Yoon et al., 2021); thus, more studies are required to disentangle the potential advantage of these approaches, and the development of robust, quantitative assays is necessary to evaluate the plasma best suited for therapeutic infusion in patients with COVID-19.

After study selection, we realized that in 4 of the selected manuscripts (Budhiraja et al., 2021; Horby et al., 2021; Joyner et al., 2021; Yoon et al., 2021), authors have performed a stratification based on the age of plasma recipients: 2 found a decreased efficiency against mortality of CP with increasing age of the recipient (Joyner et al., 2021; Yoon et al., 2021) and another found the most consistent benefit in the range 60–74 years (Budhiraja et al., 2021); the remaining manuscript, *i.e.* the only RCT containing such comparison, showed a non-significant trend toward a possible benefit in patients >80 years (Horby et al., 2021). While age may turn to be another critical parameter (in addition to disease severity) to select the most suitable patients for this treatment, more data and more studies are required to reach a conclusion.

Another hurdle in the development of this therapy may become the diminished activity of CP of a donor infected with a specific SARS-CoV-2 variant against other circulating viral lineages. The variant named 501Y.V1 (also known as B.1.1.7), which arose in the UK at the end of 2020 harboring 9 amino acid changes in the spike and showing increased transmission, was not observed to escape from antibodies generated by natural infection (Supasa et al., 2021; Wang et al., 2021b). On the other hand, the variant named 501Y.V2 (also known as B.1.351, characterized by substitutions in two immunodominant domains of the spike protein) dominated the infections of the second wave in South Africa and it was effectively neutralized by plasma from individuals who were infected during the second wave but only poorly cross-neutralized by plasma from individuals with first-wave infections (Cele et al., 2021; Wibmer et al., 2021). Indeed, CP from patients infected with SARS-CoV-2 from early in the pandemic showed a remarkable reduction of efficacy against B.1.351 variant, threatening the protective efficacy of CP -based therapies (Wang et al., 2021b). Another emergent variant from Brazil, P.1, in which a change in conformation of one of the receptor-binding domains is known to facilitate ACE2 binding, was also more resistant to neutralization by CP (Wang et al., 2021a). These cases of concern may make necessary to select therapeutic plasma with specific desired functionalities (Morgenlander et al., 2021).

In conclusion, this meta-analysis does foster a paradigm switch in the design of further RCTs, suggesting that inclusion of patients with early stage of the disease may be critical to maximize the efficacy of this therapy on COVID-19-induced mortality. Of note, these considerations are also relevant for upcoming therapies with pooled anti-SARS-CoV-2 plasma-isolated IgGs (Stasi et al., 2020), given that the rational underpinning their use is substantially overlapping, and may help to better design this practice which, importantly, may be affordable also in developing countries.

### Limitations of the study

The present results should be considered exploratory, rather than conclusive. In particular, the fact that the benefit in terms of mortality for RCTs enrolling patients with mild disease or providing treatment within 3 days of hospitalization was not statistically significant possibly depends on the reduced amount of studies and overall still low numbers of patients evaluated. In addition, given the emergency nature of the pandemic and the need for life-saving therapies with demonstrated benefit, we opted for a pragmatic approach, by extracting only one outcome and by categorizing studies according to the population enrolled and the methodology used to screen plasma samples. This method was forced by the lack of subgroups data from the collected studies, with the exception of 5 manuscripts (Alsharidah et al., 2021; Donato et al., 2021; Horby et al., 2021; Joyner et al., 2021; Salazar et al., 2020a). Another study limitation has been that, although at a qualitative level it seems reasonable to hypothesize that plasma therapy may be of better support in case of subjects with less efficacious immune system (older compared with younger SARS-CoV-2-infected patients), the reduced number of studies and the heterogeneous stratification (based on different age threshold) made a quantitative analysis of age effect unfeasible. Finally, the criteria used for manuscript categorization, albeit pre-specified and rationale-driven, are empirical. Thus, we cannot infer the exact timing, disease stage, or antibody titer making CP an effective therapy nor we can explore whether setting different criteria would have yielded different results.

## STAR★Methods

### Key resources table

**Table:** 

REAGENT or RESOURCE	SOURCE	IDENTIFIER
**Deposited data**		

Systematic review protocol	This paper	Prospero: CRD42021236146

**Software and Algorithms**		

Review manager 5.4.1	Cochrane Collaboration, London, UK	https://www.cochranelibrary.com

### Resource availability

#### Lead contact

Request for further information should be directed to and will be fulfilled by the lead contact, Prof. Giuseppe Matarese (giuseppe.matarese@unina.it)

#### Materials availability

This study did not generate new unique reagents.

#### Data and code availability

• Our systematic review protocol has been registered at the International Prospective Register of Systematic Reviews (Prospero). The study reference is listed in the key resources table.
• This paper does not report original code.
• Any additional information required to reanalyze the data reported in this paper is available from the lead contact upon request.

### Method details

#### Literature search and study selection

We searched through PubMed, Embase, Scopus and the Cochrane database up to March 31^st^, 2021, with no language restriction. We used the terms “COVID-19” {MeSH Terms}, “SARS-CoV-2” {MeSH Terms}, “convalescent plasma” {All Fields}, “plasma therapy” {All Fields}, “mortality” {All Fields}, and “death” {All Fields} as keywords. As example for the search strategy, the strings used in PubMed are also attached as supplemental information. We also scrutinized the reference lists of previous meta-analyses and included studies (Chai et al., 2020; Klassen et al., 2020; Sarkar et al., 2021). Since COVID-19 pandemic is a rapidly evolving situation, we also considered non-peer reviewed data in Clinicaltrial.gov and in a preprint repository, *i.e.* medRxiv.org. Overall, 753 abstracts were reviewed.

Two investigators (F.P. and P.d.C.) independently reviewed the identified abstracts to determine the eligibility of the studies for inclusion in the meta-analysis. Eligibility criteria for all studies were: 1 - enrolling patients with laboratory-confirmed COVID-19; 2 - treating patients with plasma obtained from patients recovered from COVID-19; 3 - unequivocal reporting of days from symptoms onset or hospitalization to treatment and clear description of the severity of the disease in patients receiving the treatment; 4 - clear description of the methodology used to test, select, or characterize plasma samples to be transfused (pre- or post-procedure); 5 - reporting data on mortality at any follow-up length. No restriction was posed for the comparative treatment nor for the follow-up length. Exclusion criteria were: 1 - reporting data from an exiguous number of subjects (*i.e*. <10 enrolled patients); 2 - missing mortality data; 3 - insufficient detailing of enrolling criteria, timing of the treatment, characterization of transfused plasma, and description of the comparative group. The only endpoint considered was all-cause mortality at any follow-up length. In case of multiple follow-up points, data from the latest time point were extracted. Discrepancies regarding the inclusion of specific manuscripts were resolved by the senior authors (A.C. and G.M.). The protocol is available on Prospero (CRD42021236146) and registered in osf.io (https://osf.io/kvqj3). The PRISMA checklist is attached in the supplemental information.

#### Data extraction and quality assessment

A pre-specified, standardized collection form (Excel sheet) was used to extract general and summary estimates data of included studies. Collected information were study type, number of patients in each group, age and sex of the patients enrolled, comparison treatment, severity of the disease, median days form symptoms onset or hospital admission to treatment, the origin of plasma (if it was from a single patient or pooled from multiple patients), whether the plasma was checked for virus neutralization activity *in vitro* and/or for antibody titer (both parameters evaluated either pre- or post-intervention) and, eventually, the titers used, and days of the longest follow-up with the corresponding risk ratio (RR) of mortality along with the 95% confidence interval (CI). Data were independently extracted by two authors (F.P. and P.d.C) and checked for accuracy by one additional investigator (S.G.). Two authors (F.P and S.G.) independently assessed the quality of included studies at outcome level (mortality) using the Risk of bias 2.0 and the ROBINS-I tools for RCTs and non-randomized studies, respectively. Discrepancies regarding the evaluation of selected items were resolved by a third author (P.d.C.).

### Quantification and statistical analysis

The RR extracted from the studies were used to calculate log RR with 95% CI for every item. When RR were not provided, they were calculated from the number of crude events. We used the Inverse Variance statistical method with RR as effect measure to test the effect of convalescent plasma on the endpoint mortality. Fixed or random effects were applied as analysis model depending on the heterogeneity across studies. Statistical heterogeneity between trials was evaluated by I^2^ statistics. Significance for heterogeneity was set at I^2^ > 50% or p < 0.1, in which case the random-effects model was used for analysis. At p > 0.1 and I^2^ < 50%, we considered heterogeneity as insignificant and the fixed-effects model was used. Different meta-analyses were performed according to different scenarios with the relative, pre-specified rationale, as detailed in the results. This approach was selected instead of subgroup analyses since none of the collected studies reported data for patients' subgroups. Analyses were performed using review manager 5.4.1 (Cochrane Collaboration, London, United Kingdom).

## Footnotes

Supplemental information can be found online at https://doi.org/10.1016/j.isci.2021.102898.
