# Supplementary material for: Effect of time and titer in convalescent plasma therapy for COVID-19
Source: iScience. 2021 Jul 22;24(8):102898. doi: 10.1016/j.isci.2021.102898 (PMC8297982; doi:10.1016/j.isci.2021.102898)
Supplement: Document S1. Figures S1–S4 and Tables S1 and S2 [file mmc1.pdf]

iScience, Volume *24*

Supplemental information

Effect of time and titer in convalescent plasma therapy for COVID-19

Paola de Candia, Francesco Prattichizzo, Silvia Garavelli, Rosalba La Grotta, Annunziata De Rosa, Agostina Pontarelli, Roberto Parrella, Antonio Ceriello, and Giuseppe Matarese

Supplemental Figure 1. Inclusion flow for literature search and study selection, related to Figures 1-3.

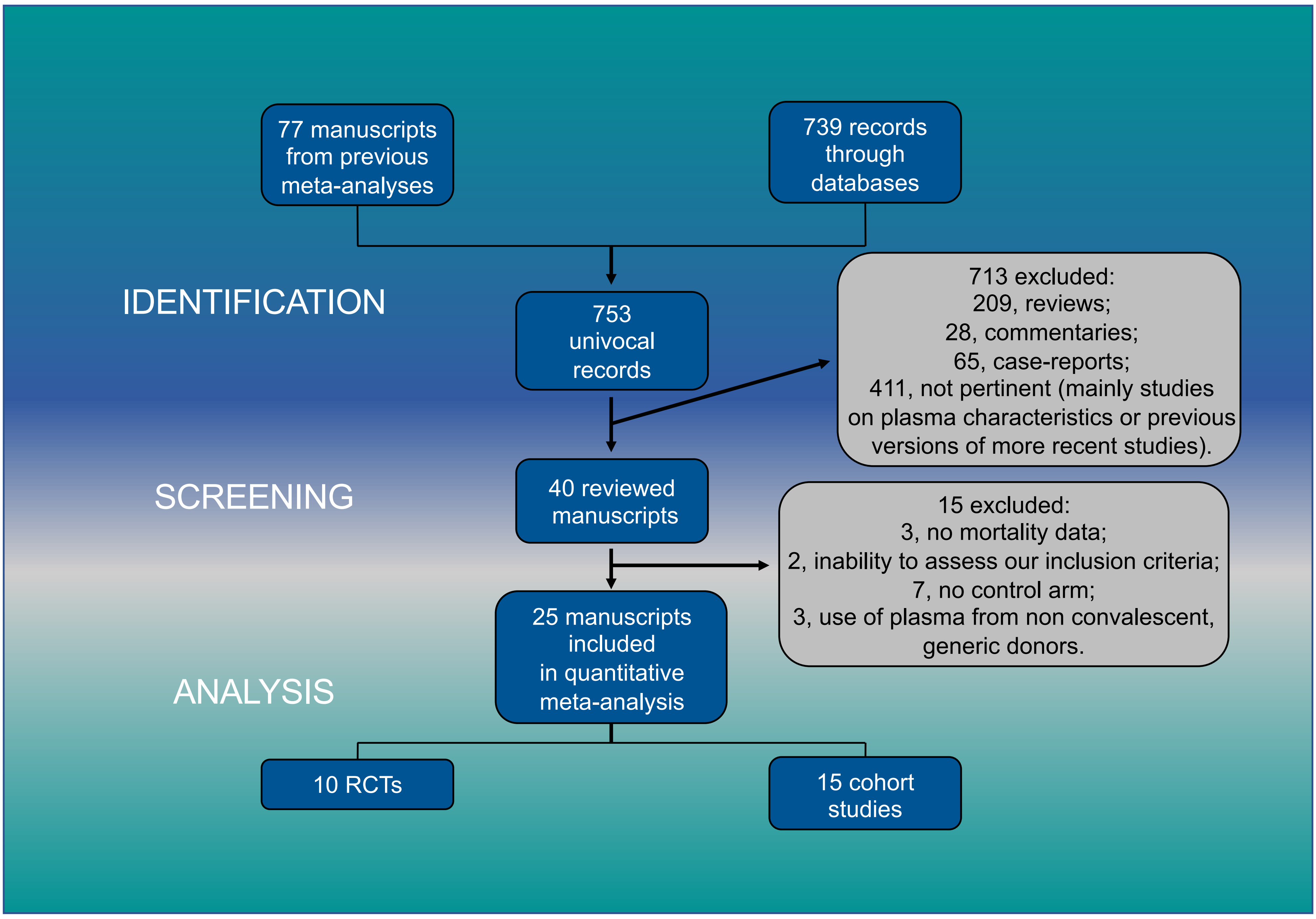

Supplemental Figure 2. Funnel plot to assess publication bias, related to Figures 1-3.

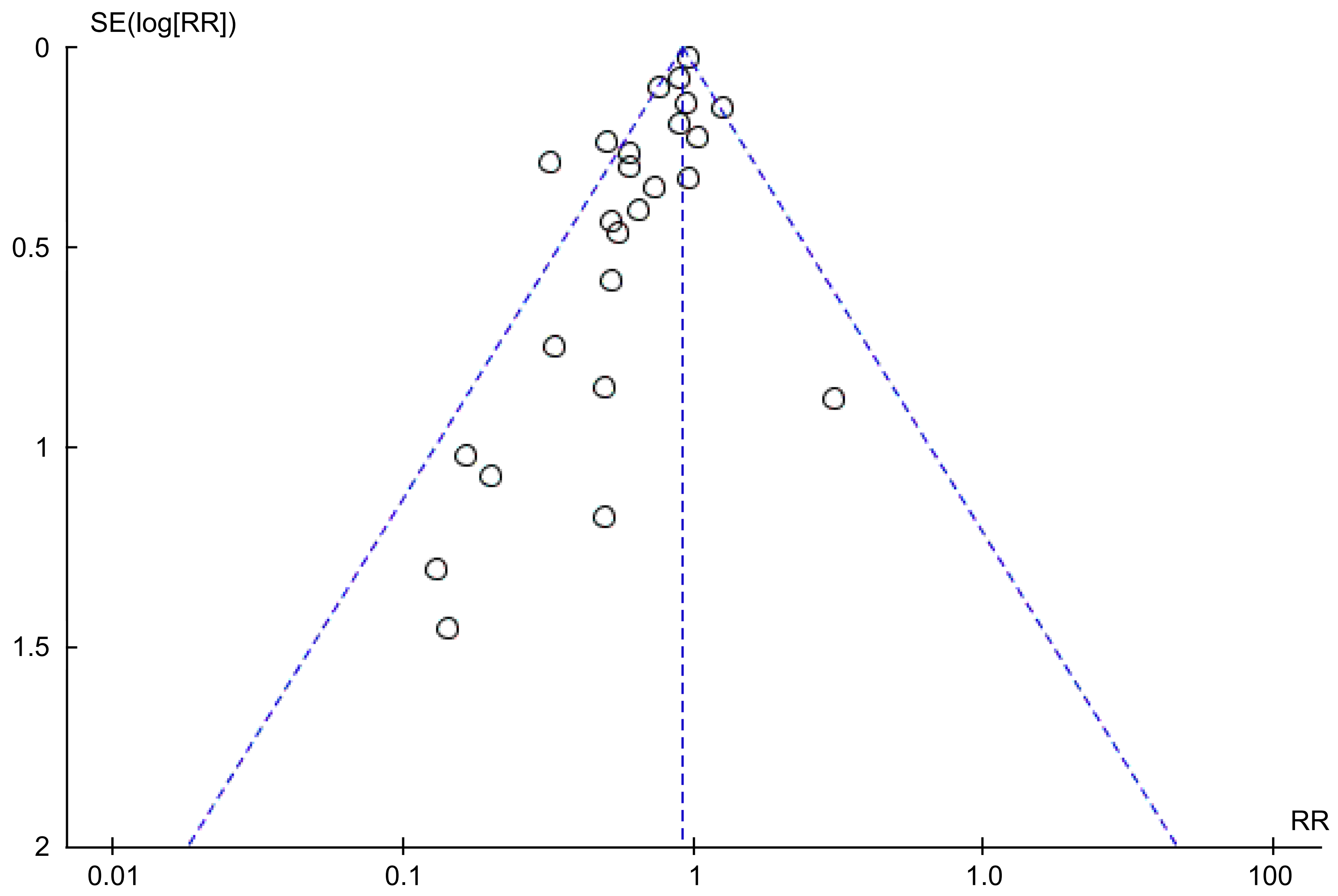

Supplemental Figure 3. Sensitivity analysis excluding the studies with different design and those falling outside the 95% CI in the funnel plot, relative to Figure 1.

Study or Subgroup	log[Risk Ratio]	SE	Weight	Risk Ratio IV, Fixed, 95% CI
Abolghasemi *et al.*, 2020	-0.498	0.3036	0.8%	0.61 [0.34, 1.10]
Agarwal *et al.*, 2020	0.0392	0.2313	1.4%	1.04 [0.66, 1.64]
Al Qahtani *et al.*, 2020	-0.6931	1.1748	0.1%	0.50 [0.05, 5.00]
Altuntas *et al.*, 2021	-0.1163	0.0799	12.1%	0.89 [0.76, 1.04]
Avendano-Sola *et al.*, 2020	-2.0402	1.3087	0.0%	0.13 [0.01, 1.69]
Budhiraja *et al.*, 2021	0.239	0.1535	3.3%	1.27 [0.94, 1.72]
Donato *et al.*, 2021	-0.5032	0.2682	1.1%	0.60 [0.36, 1.02]
Duan *et al.*, 2020	-1.9459	1.4505	0.0%	0.14 [0.01, 2.45]
Gharbharan *et al.*, 2020	-0.5978	0.4675	0.4%	0.55 [0.22, 1.38]
Hegerova *et al.*, 2020	-1.0986	0.7528	0.1%	0.33 [0.08, 1.46]
Li *et al.*, 2020	-0.4308	0.4118	0.5%	0.65 [0.29, 1.46]
Libster *et al.*, 2021	-0.6931	0.8515	0.1%	0.50 [0.09, 2.65]
Liu *et al.*, 2020	-0.6419	0.4408	0.4%	0.53 [0.22, 1.25]
Omrani *et al.*, 2021	-1.6094	1.0724	0.1%	0.20 [0.02, 1.64]
Rasheed *et al.*, 2020	-1.7918	1.0206	0.1%	0.17 [0.02, 1.23]
RECOVERY trial, 2021	-0.0305	0.0326	72.4%	0.97 [0.91, 1.03]
Shenoy *et al.*, 2021	-0.058	0.1463	3.6%	0.94 [0.71, 1.26]
Simonovich *et al.*, 2020	-0.0408	0.3328	0.7%	0.96 [0.50, 1.84]
Xia *et al.*, 2020	-0.6407	0.5851	0.2%	0.53 [0.17, 1.66]
Yoon *et al.*, 2021	-0.3011	0.3537	0.6%	0.74 [0.37, 1.48]
Zeng *et al.*, 2020	-0.1133	0.1952	2.0%	0.89 [0.61, 1.31]
**Total (95% CI)**			**100.0%**	**0.94 [0.89, 1.00]**

Heterogeneity: $Chi^2 = 27.20$, df = 20 ($p = 0.13$); $I^2 = 26\%$
Test for overall effect: Z = 2.11 ($p = 0.04$)

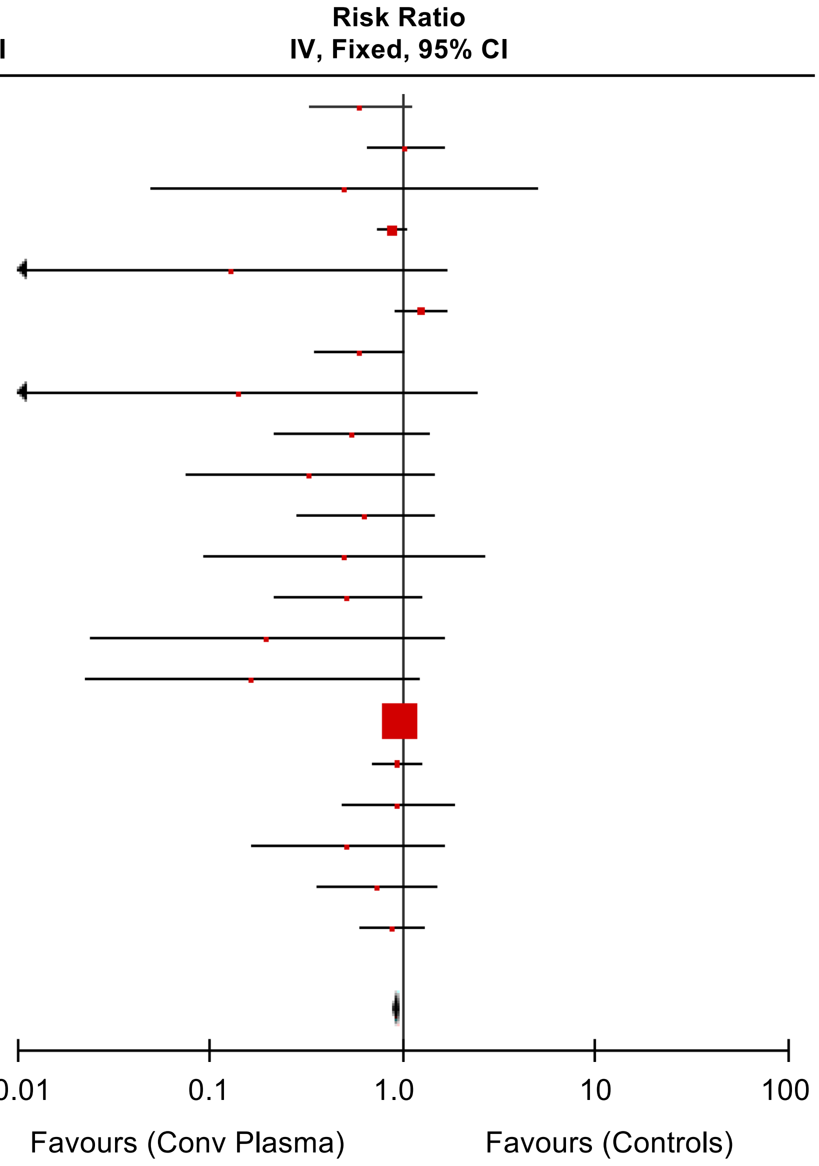

Supplemental Figure 4. Forest plots summarizing the effect of convalescent plasma vs. standard of care or placebo or no treatment on mortality incidence considering only the specified studies, relative to Figures 2 and 3.

A. Only RCTs

Study or Subgroup	log[Risk Ratio]	SE	Weight	Risk Ratio IV, Fixed, 95% CI
Agarwal *et al*., 2020	0.0392	0.232	1.9%	1.04 [0.66, 1.64]
Al Qahtani *et al*., 2020	-0.6931	1.1748	0.1%	0.50 [0.05, 5.00]
Avendano-Sola *et al*., 2020	-2.0402	1.3087	0.1%	0.13 [0.01, 1.69]
Balcells *et al*., 2021	1.1119	0.8817	0.1%	3.04 [0.54, 17.12]
Gharbharan *et al*., 2020	-0.5978	0.4675	0.5%	0.55 [0.22, 1.38]
Li *et al*., 2020	-0.4308	0.4118	0.6%	0.65 [0.29, 1.46]
Libster *et al*., 2021	-0.6931	0.8515	0.1%	0.50 [0.09, 2.65]
Rasheed *et al*., 2020	-1.7918	1.0206	0.1%	0.17 [0.02, 1.23]
RECOVERY trial, 2021	-0.0305	0.0326	95.6%	0.97 [0.91, 1.03]
Simonovich *et al*., 2020	-0.0408	0.3328	0.9%	0.96 [0.50, 1.84]
**Total (95% CI)**			**100.0%**	**0.96 [0.91, 1.03]**

Heterogeneity: Chi^2 =10.40, df = 9 (p = 0.32); I^2 = 13%
Test for overall effect: Z = 1.17 (p = 0.24)

Risk Ratio
IV, Fixed, 95% CI

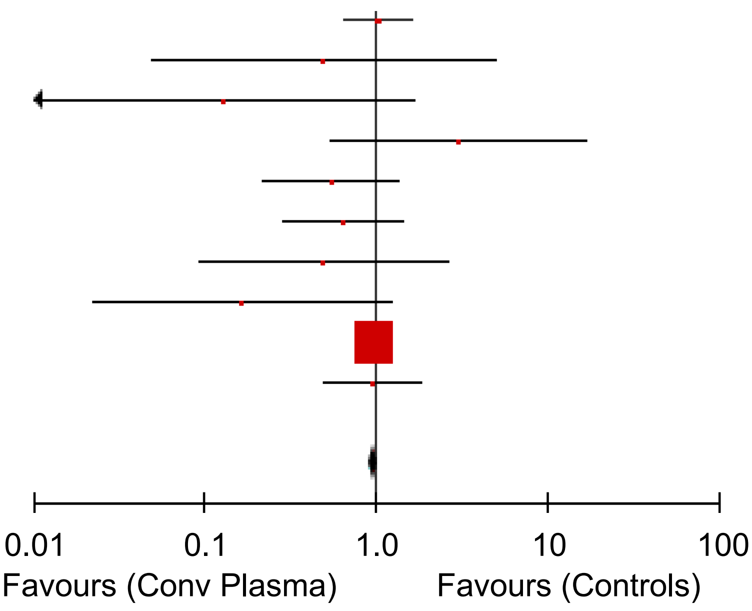

B. Only RCTs enrolling patients with mild disease or providing treatment within 3 days of hospitalization

Study or Subgroup	log[Risk Ratio]	SE	Weight	Risk Ratio IV, Fixed, 95% CI
Avendano-Sola *et al*., 2020	-2.0402	1.3087	1.7%	0.13 [0.01, 1.69]
Balcells *et al*., 2021	1.1119	0.8817	3.8%	3.04 [0.54, 17.12]
Libster *et al*., 2021	-0.6931	0.8515	4.0%	0.50 [0.09, 2.65]
Rasheed *et al*., 2020	-1.7918	1.0206	2.8%	0.17 [0.02, 1.23]
RECOVERY trial, 2021	-0.1863	0.1829	87.7%	0.83 [0.58, 1.19]
**Total (95% CI)**			**100.0%**	**0.79 [0.57, 1.11]**

Heterogeneity: Chi^2 =6.92, df = 4 (p = 0.14); I^2 = 42%
Test for overall effect: Z = 1.37 (p = 0.17)

Risk Ratio
IV, Fixed, 95% CI

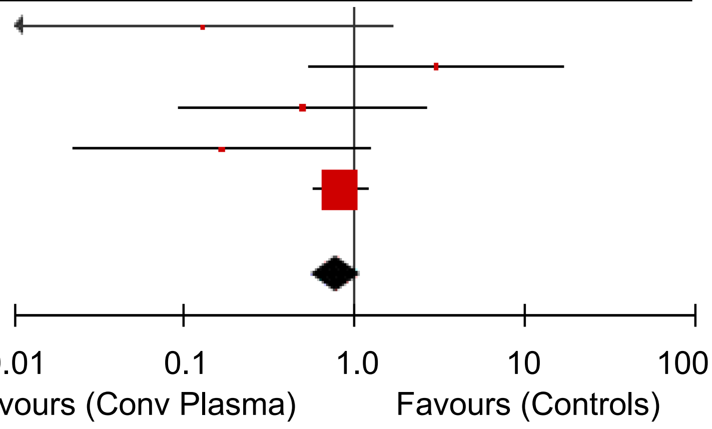

C. Only RCTs enrolling patients with severe disease or providing treatment after 3 days of hospitalization

Study or Subgroup	log[Risk Ratio]	SE	Weight	Risk Ratio IV, Fixed, 95% CI
Agarwal *et al*., 2020	0.0392	0.232	16.2%	1.04 [0.66, 1.64]
Al Qahtani *et al*., 2020	-0.6931	1.1748	0.6%	0.50 [0.05, 5.00]
Gharbharan *et al*., 2020	-0.5978	0.4675	4.0%	0.55 [0.22, 1.38]
Li *et al*., 2020	-0.4308	0.4118	5.1%	0.65 [0.29, 1.46]
RECOVERY trial, 2021	0.174	0.1149	66.1%	1.19 [0.95, 1.49]
Simonovich *et al*., 2020	-0.0408	0.3328	7.9%	0.96 [0.50, 1.84]
**Total (95% CI)**			**100.0%**	**1.07 [0.89, 1.29]**

Heterogeneity: Chi^2 =4.89, df = 5 (p = 0.43); I^2 = 0%
Test for overall effect: Z = 0.73 (p = 0.47)

Risk Ratio
IV, Fixed, 95% CI

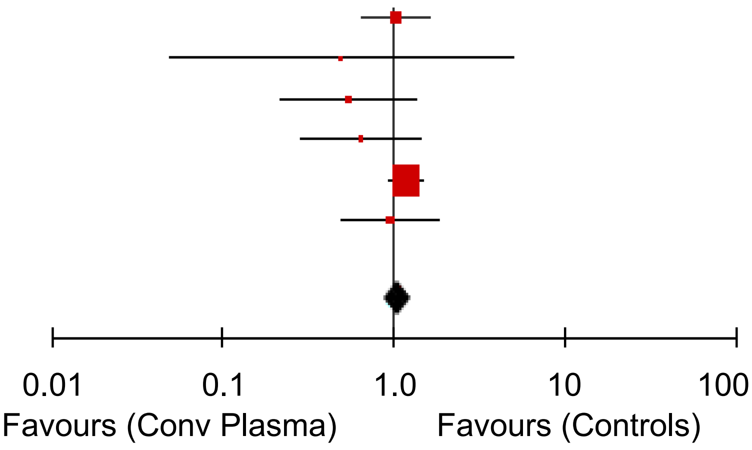

D. Only RCTs checking the presence of antibody without posing a cut-off to select convalescent plasma samples or with no check

Study or Subgroup	log[Risk Ratio]	SE	Weight	Risk Ratio IV, Fixed, 95% CI
Agarwal *et al*., 2020	0.0392	0.2313	91.7%	1.04 [0.66, 1.64]
Al Qahtani *et al*., 2020	-0.6931	1.1748	3.6%	0.50 [0.05, 5.00]
Rasheed *et al*., 2020	-1.7918	1.0206	4.7%	0.17 [0.02, 1.23]
**Total (95% CI)**			**100.0%**	**0.93 [0.60, 1.43]**

Heterogeneity: Chi^2 =3.35, df = 2 (p = 0.19); I^2 = 40%
Test for overall effect: Z = 0.33 (p = 0.74)

Risk Ratio
IV, Fixed, 95% CI

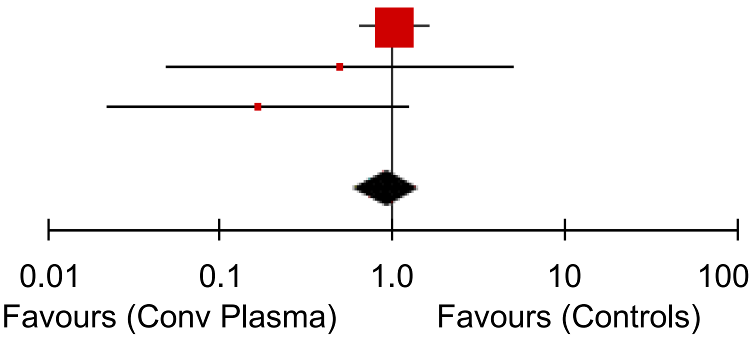

E. Only RCTs using high antibody-titer convalescent plasma

Study or Subgroup	log[Risk Ratio]	SE	Weight	Risk Ratio IV, Fixed, 95% CI
Avendano-Sola *et al*., 2020	-2.0402	1.3087	1.1%	0.13 [0.01, 1.69]
Balcells *et al*., 2021	1.1119	0.8817	2.4%	3.04 [0.54, 17.12]
Gharbharan *et al*., 2020	-0.5978	0.4675	8.7%	0.55 [0.22, 1.38]
Li *et al*., 2020	-0.4308	0.4118	11.2%	0.65 [0.29, 1.46]
Libster *et al*., 2021	-0.6931	0.8515	2.6%	0.50 [0.09, 2.65]
RECOVERY trial, 2021	-0.1863	0.1829	56.8%	0.83 [0.58, 1.19]
Simonovich *et al*., 2020	-0.0408	0.3328	17.2%	0.96 [0.50, 1.84]
**Total (95% CI)**			**100.0%**	**0.80 [0.61, 1.04]**

Heterogeneity: Chi^2 =5.76, df = 6 (p = 0.45); I^2 = 0%
Test for overall effect: Z = 1.64 (p = 0.10)

Risk Ratio
IV, Fixed, 95% CI

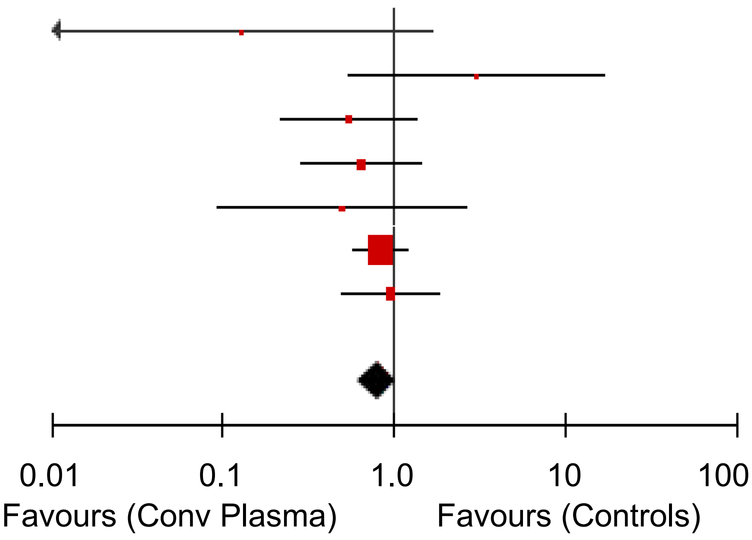

Supplemental Table 1. Patients' characteristics and data extracted from the manuscripts included in the quantitative meta-analysis, related to Figures 1-3.

Study ID	Study type	Number of patients enrolled (Conv Plasma/Controls)	Control treatment	Characteristics of patients enrolled	Median time from admission to treatment	Classification Early/Late	Sex N (%)	Age (median, IQR or mean ± SD)	Single donor or pooled plasma	Severity of the disease in donors and inclusion criteria	Checking virus neutralization *in vitro* (yes/no)	Cut-off to select high-titer plasma (yes/no and value)	Classification as high titer vs no check or positive only	Longest follow-up (days)	Risk Ratio for all-cause mortality at longest follow-up	Reference
Agarwal et al., 2020	Randomized clinical trial	235/229	Standard of care	Severe	Not provided	Late	**CP**: F 58 (25); M 177 (75)<br>**Control group**: F 52 (23) M 177 (77)	**CP**: 52 (42-60)<br>**Control group**: 52 (41-60)	2 doses from different donors	Negative at RT-PCR for 2 weeks or 4 weeks without syntoms	No	IgG titer from 1:20 to 1:80 in a minor portion of the samples (subgroup data only for primary outcome and not for mortality)	Positive only	28 days	1.04 [0.66, 1.64]	28
Al Qahtani et al., 2020	Randomized clinical trial	20/20	Standard therapy	Severe	Not provided	Late	**CP**: F 3 (15); M 17 (85)<br>**Control group**: F 5 (25) M 15 (75)	**CP**: 52.6 ± 14.9<br>**Control group**: 50.7 ± 12.5	Not provided	Donors discharged from hospital for more than 2 weeks	No	Only tested for IgM/IgG presence	Positive only	Days not specified	0.50 [0.05, 5.00]	29
Avendano-Sola et al., 2020	Randomized clinical trial	38/43	Standard of care	Mild	8 days	Early	**CP**: F 18 (47.4); M 20 (52.6)<br>**Control group**: F 19 (44.2) M 24 (55.8)	**CP**: 61.3 ± 16.3<br>**Control group**: 60.3 ± 15	Single donor	Asymptomatic for at least 14 days	Viral microneutralization test (*a posteriori*)	ID50-1:80	High titer	29 days	0.13 [0.01, 1.69]	30
Balcells et al., 2021	Randomized clinical trial	Early plasma: n=28<br>Deferred plasma: n=30	No control group<br>Sugroups of comparison: immediate CP (early) *vs* no CP unless deterioration (deferred treatment)	Moderate but at high risk of developing complications	≥7 days of symptoms	Early and late subgroup data	**Early plasma:** F 13 (46.4); M 15 (53.6)<br>**Deferred plasma:** F 16 (53.3); M 14 (46.7)	**Early plasma:** 64.3 (33-92)<br>**Deferred plasma:** 67.1 (27-91)	Not specified	History of symptomatic, but not severe, COVID-19; asymptomatic for at least 28 days and negative RT-qPCR from swab and plasma	No	Antibody titer ≥1:400	High titer	30 days	3.04 [0.54–17.17]	36
Gharbharan et al., 2020	Randomized clinical trial	43/43	Standard of care	Moderate to severe	Not provided	Not classifiable	**CP**: F 14 (33); M 29 (67)<br>**Control group**: F 10 (23) M 33 (77)	**CP**: 61 (56-70)<br>**Control group**: 63 (55-77)	Single donor	Asymptomatic for at least 14 days	No	Antibody titer ≥ 1:80	High titer	60 days	0.55 [0.22, 1.38]	31
Libster et al., 2021	Randomized clinical trial	80/80	Placebo (saline solution)	Mild	72 hours	Early	**CP**: F 54 (68); M 26 (32)<br>**Control group**: F 46 (58) M 34 (42)	**CP**: 76.4 ± 8.7<br>**Control group**: 77.9 ± 8.4	Single donor	SARS-CoV-2 infection for at least 10 days, asymptomatic for 3 days and negative for two RT-PCR tests	No	Antibody titer ≥ 1:1000	High titer	25 days	0.50 [0.09, 2.65]	32
Li et al., 2020	Randomized clinical trial	52/51	Standard of care	Severe	72 hours	Late	**CP**: F 25 (48.1); M 27 (51.9)<br>**Control group**: F 18 (35.3) M 33 (64.7)	**CP**: 70 (62-80)<br>**Control group**: 69 (63-76)	Single donor	Negative for two RT-PCR tests and discharged for more than 2 weeks	No	Antibody titer ≥ 1:640	High titer	28 days	0.65 [0.29, 1.46]	33
Rasheed et al., 2020	Randomized clinical trial	21/28	Standard of care	Moderate to Severe	3 days	Early	**CP**: F 9 (42.9); M 12 (57.1)<br>**Control group**: not provided	**CP**: 55.6 ± 17.83<br>**Control group**: 47.82 ± 15.36	Not provided	Moderate COVID-19 infections	No	IgG index ≥1.25	Positive only	5 days	0.17 [0.02, 1.23]	34
RECOVERY trial, 2021	Randomized clinical trial	5795/5763	Standard of care	Moderate to severe	2 days	Early and late subgroup data	**CP**: F 2152 (37); M 3643 (63)<br>**Control group**: F 1976 (34) M 3787 (66)	**CP**: 63.6 (SD14.7)<br>**Control group**: 63.4 (SD14.6)	Mostly from 2 donors	Not provided	No	Antibody titer ≥ 1:100	High titer	28 days	0.97 [0.91, 1.03]	37
Simonovich et al., 2020	Randomized clinical trial	228/105	Placebo (saline solution)	Severe	7 days	Late	**CP**: F 67 (29.4); M 161 (70.6)<br>**Control group**: F 41 (39) M 64 (61)	**CP**: 62.5 (53-72.5)<br>**Control group**: 62 (49-71)	Single and pooled (from 2 to 5) donors	Not provided	Yes	Antibody titer ≥ 1:400	High titer	30 days	0.96 [0.50, 1.84]	35
Abolghasemi et al., 2020	Prospective cohort study	115/74	Standard of care	Moderate to severe	7 days	Late	**CP**: F 48(41.7); M 67 (58.3)<br>**Control group**: F 37 (50) M 37 (50)	**CP**: 54.41 ± 13.71<br>**Control group**: 56.83 ± 14.68	Not provided	No syntomps for 14 day, negative at RT-PCR	No	Antibody titer cutoff index>1.1	Positive only	Days not specified	0.61 [0.34, 1.10]	38
Alsharidah et al. 2021	Prospective cohort study	135/233	Standard of care	Patients stratified into moderate (early) and severe (late)	24 hours	Early and late subgroup data	**CP**: F 30 (22.2); M 105 (77.8)<br>**Control**: F 35 (15); M 198 (85)	**CP**: 54 (48-60)<br>**Control group**: 54 (45-62)	Not provided	Not provided	No	No	Positive only	30 days	moderate: 0,27 [0,12–0,62]<br>severe: 0,38 [0,14–1,02]	45
Altuntas et al. 2021	Retrospective cohort study	888/888	favipravir, lopinavir + ritonavir, hydroxychloroquine, high dose vitamin C, azithromycin	Severe or critical	≤5; 6-10; 11-15; 16-20; >20 days from symptom onset	Late	**CP**: F272 (30,6); M 616 (69,4)<br>**Control group:** F 254 (28,6); M 634 (71,4)	**CP**: 60 (19-96)<br>**Control group**: 61 (21-91)	Not provided	Resolution of symptoms at least 14 days prior to donation and negative results for COVID-19	No	No	Positive only	Days not specified	0.89 [0.76, 1.04]	48
Budhiraja et al., 2021	Retrospective cohort study	333/361	Standard of care	Moderate to severe	Not provided	Late	**CP**: F 66 (19.8); M 267 (80.2)<br>**Control group:** F 100 (27.7); M 261 (72.3)	**Entire cohort**:<br><45: 40 (CP); 62 (control)<br>45-59: 122 (CP); 115 (control)<br>60-74: 135 (CP); 149 (control)<br>75+: 36 (CP); 35 (control)	Single donor	Either complete resolution of symptoms 28 days or 14 days and two negative swabs before donation	No	Low titer=15-80 AU/ml<br>High titer> 80 AU/ml	Mixed: either positive only or high titer depending on the centre	28 days	1.27 [0.94, 1.72]	47
Donato et al., 2021	Observational cohort study	Not requiring ventilation:36<br>Requiring ventilation: 15	Network database for hospitalized patients with COVID-19 pneumonia	Moderate to severe	7 days	Early and late subgroup data	**Not requiring ventilation:** F 21 (58.3); M 15 (41.7)<br>**Requiring ventilation:** F 5 (33); M 10 (67)	**Not requiring ventilation:** 58 (IQR 49–68)<br>**Requiring ventilation** 53 (IQR 45–58)	Not provided	At least 14 days from resolution of symptoms and 1 subsequent negative swab	Yes	IgG titer >1:500	High titer	30 days	0.60 [0.36, 1.02]	46
Duan et al., 2020	Retrospective cohort study	10/10	Standard of care	Severe	6 days	Late	**CP**: F 4 (40); M 6 (60)<br>**Control group:** F 4 (40); M 6 (60)	**CP**: 52.5 (45-59.5)<br>**Control group**: 53 (46.5-60.5)	Single donor	3 weeks post disease onset and 20 days post-discharging	No	Antibody titer >1:640	High titer	7 days	0.14 [0.01, 2.45]	39
Hegerova et al., 2020	Retrospective cohort study	20/20	Standard of care	Moderate to severe	2 days	Early	**CP**: not provided<br>**Control group**: not provided	**CP**: 60 (29-95)<br>**Control group**: not provided	Single donor	>28 days past last symptom	No	No quantification	No check	14 days	0.33 [0.08, 1.46]	40
Joyner et al., 2020	Retrospective cohort study	Low titer: n=561<br>Medium titer: n=2006<br>High titer: n=515	No control group<br>Sugroup of comparison: high titer vs low titer; early treatment vs late treatment	Moderate to severe	Early treatment <3 days<br>Late treatment >3 days	Early and late subgroup data	**Low titer**: F 201 (36.0); M 357 (60.0); undisclosed 1; unreported 2<br>**Medium titer**: F 774 (38.7); M 1227 (61.3); undisclosed 1; unreported 4<br>**High titer**: F 221 (43.0); M 293 (57.0); undisclosed 1	**Entire cohort**:<br>18-39 yrs: 267<br>40-59 yrs: 1057<br>60-69 yrs: 798<br>70-79 yrs: 606<br>≥80 yrs: 354	One or more donor (not pooled)	Not provided	No	Low titer<4.62<br>Medium titer 4.62 to 18.45<br>High titer> 18.65	Subgroup data for high titer	30 days	0.77 [0.63, 0.94]	13
Liu et al., 2020	Retrospective cohort study with propensity-matching	39/156	Standard of care	Severe	4 days	Late	**CP**: F 14 (36); M 25 (64)<br>**Control group**: F 45 (28.9); M 111 (71.1)	**CP**: 55 ± 13<br>**Control group**: 56 ± 14	Single donor	Not provided	No	Antibody titer ≥ 1:320	High titer	31 days	0.53 [0.22, 1.25]	41
Omrani et al., 2021	Retrospective cohort study	40/40	Standard of care	Mostly requiring intensive ventilation	Within the first 7 days from admission to intensive care unit	Late	**CP:** F 6 (15); M 34 (85)<br>**Control group**: F 5 (12.5); M 35 (87.5)	**CP:** 47.5 (39-60.5) **Control group**: 55.5 (46.5-60.5)	Not specified	Mostly severe, plasma donation 26 days after documented viral clearance	No	No quantification	No check	28 days	0.20 [0.02, 1.64]	49
Salazar et al., 2021	Prospective cohort study with propensity-matching	321/582	Subgroup of comparison: high titer vs no treatment; early treatment vs no treatment	Moderate to severe	Early treatment <3 days<br>Late treatment >3 days	Early and late subgroup data	**CP**: F 137 (42.7); M 184 (57.3)<br>**Control group**: F 258 (44.3); M 324 (55.7)	**Entire cohort:**<br><30 yrs: 36<br>30-39 yrs: 114<br>40-49 yrs: 175<br>50-59 yrs: 242<br>60-69 yrs: 212<br>70-79 yrs: 89<br>≥80 yrs: 35	Single donor	Asymptomatic for more than 14 days	No	Antibody titer ≥ 1:1350	Subgroup data for high titer	28 days	0.50 [0.31, 0.81]	42
Shenoy et al., 2020	Retrospective cohort study	263/263	Standard of care	Severe or critical	Not provided	Late	**CP**: F 96 (36.5); M 167 (63.5)<br>**Control group**: F 96 (36.5) M 167 (63.5)	**CP**: 55.93 (SD 14.01)<br>**Control group**: 56.1 (SD 14.00)	Not specified	Not provided	No	No quantification	No check	28 days	0.94 [0.71, 1.26]	50
Xia et al., 2020	Retrospective cohort study	138/1430	Standard of care	Severe	45 days	Late	**CP**: F 61(44.2); M 77 (55.8)<br>**Control group**: F 710 (49.7) M 720 (50.3)	**CP**: 65 (57-73)<br>**Control group**: 63 (53-71)	Not provided	3 weeks after symtomps onset	No	Antibody titer ≥ 1:160	High titer	21 days	0.53 [0.17, 1.66]	43
Yoon et al., 2021	Retrospective cohort study with propensity-matching	73/73	Standard of care	Severe	72 hours	Early	**CP**: F 32 (43.8); M 41 (56.2)<br>**Control group**: F 26 (35.6) M 47 (64.4)	**CP**: 67 (55-75)<br>**Control group**: 66 (56-77)	Pooled	Not provided	Yes	Antibody titer ≥ 1:2430	High titer	28 days	0.74 [0.37–1.46]	51
Zeng et al., 2020	Retrospective cohort study	6/15	Standard of care	Severe	21.5 days	Late	**CP**: F 1(16.7); M 5 (83.3)<br>**Control group**: F 4 (26.7) M 11 (73.3)	**CP**: 61.5 (31.5-77.8)<br>**Control group**: 73 (60-79)	Not provided	1 or 2 weeks without symptoms and negative for RT-PCR test and IgM quantification	No	No quantification	No check	Observation time from 19th Feb 2020 to 1st Apr 2020	0.89 [0.61, 1.31]	44

Supplemental Table 2. Qualitative evaluation of the risk of bias of the included studies using the Risk of bias 2.0 and the ROBINS-I tools for randomized trials and non-randomized studies, respectively (green, yellow, and red colours indicate no concerns, some concerns, and serious concerns), related to Figures 1-3.

Clinical trials	Randomization process	Deviation from intended interventions	Missing data	Measurement of outcome	Selection of reported results	Overall
Agarwal *et al.*, 2020	green	green	red	green	yellow	yellow
Al Qahatani *et al.*, 2020	green	yellow	green	yellow	yellow	yellow
Avendano-Sola *et al.*, 2020	green	green	green	yellow	yellow	yellow
Balcells *et al.*, 2021	green	green	green	green	green	green
Gharbaran *et al.*, 2020	green	yellow	green	green	green	green
Horby *et al.*, 2021	green	green	green	green	green	green
Libster *et al.*, 2021	green	green	green	green	green	green
Li *et al.*, 2020	green	green	green	green	green	green
Rasheed *et al.*, 2020	red	green	green	red	red	red
Simonovich *et al.*, 2020	green	green	green	green	yellow	green

Non-randomized studies	Confounding	Selection of participants	Classification of interventions	Deviation from intended interventions	Missing data	Measurement of outcome	Selection of reported results	Overall
Abolghasemi *et al.*, 2020	yellow	red	red	green	red	red	red	red
Alsharidah *et al.*, 2021	yellow	yellow	green	green	green	green	green	yellow
Altuntas *et al.*, 2021	green	yellow	yellow	green	red	yellow	red	red

Budhiraja *et al.*, 2021	🔴	🟡	🟡	🟢	🟢	🟡	🔴	🔴
Donato *et al.*, 2021	🟡	🟡	🟢	🟢	🟢	🟢	🟢	🟡
Duan *et al.*, 2020	🔴	🔴	🔴	🟢	🔴	🔴	🔴	🔴
Hegerova *et al.*, 2020	🟡	🔴	🔴	🟢	🟢	🟡	🔴	🔴
Joyner *et al.*, 2021	🟢	🟢	🟡	🟢	🟡	🟢	🟡	🟢
Liu *et al.*, 2020	🔴	🟡	🔴	🟢	🟢	🟡	🔴	🔴
Omrani et al.,	🔴	🟢	🟡	🟢	🟡	🟡	🔴	🔴
Salazar *et al.*, 2020	🟡	🟡	🔴	🟢	🟢	🔴	🔴	🔴
Shenoy *et al.*, 2021	🟡	🟢	🟢	🟢	🟢	🟡	🟡	🟡
Xia *et al.*, 2020	🔴	🔴	🟡	🟢	🟢	🔴	🔴	🔴
Yoon *et al.*, 2021	🟢	🟡	🟡	🟢	🟢	🟡	🟡	🟢
Zeng *et al.*, 2020	🔴	🟡	🔴	🟢	🟢	🟢	🔴	🔴

PRISMA 2009 Checklist

Section/topic	#	Checklist item	Reported on page #
**TITLE**			
Title	1	Identify the report as a systematic review, meta-analysis, or both.	1
**ABSTRACT**			
Structured summary	2	Provide a structured summary including, as applicable: background; objectives; data sources; study eligibility criteria, participants, and interventions; study appraisal and synthesis methods; results; limitations; conclusions and implications of key findings; systematic review registration number.	2
**INTRODUCTION**			
Rationale	3	Describe the rationale for the review in the context of what is already known.	3
Objectives	4	Provide an explicit statement of questions being addressed with reference to participants, interventions, comparisons, outcomes, and study design (PICOS).	3-4
**METHODS**			
Protocol and registration	5	Indicate if a review protocol exists, if and where it can be accessed (e.g., Web address), and, if available, provide registration information including registration number.	Transparent methods (Supplementary)
Eligibility criteria	6	Specify study characteristics (e.g., PICOS, length of follow-up) and report characteristics (e.g., years considered, language, publication status) used as criteria for eligibility, giving rationale.	Transparent methods (Supplementary)
Information sources	7	Describe all information sources (e.g., databases with dates of coverage, contact with study authors to identify additional studies) in the search and date last searched.	Transparent methods (Supplementary)
Search	8	Present full electronic search strategy for at least one database, including any limits used, such that it could be repeated.	Transparent methods (Supplementary)
Study selection	9	State the process for selecting studies (i.e., screening, eligibility, included in systematic review, and, if applicable, included in the meta-analysis).	Transparent methods (Supplementary)
Data collection process	10	Describe method of data extraction from reports (e.g., piloted forms, independently, in duplicate) and any processes for obtaining and confirming data from investigators.	Transparent methods (Supplementary)

PRISMA 2009 Checklist

Data items	11	List and define all variables for which data were sought (e.g., PICOS, funding sources) and any assumptions and simplifications made.	Transparent methods (Supplementary)
Risk of bias in individual studies	12	Describe methods used for assessing risk of bias of individual studies (including specification of whether this was done at the study or outcome level), and how this information is to be used in any data synthesis.	Transparent methods (Supplementary)
Summary measures	13	State the principal summary measures (e.g., risk ratio, difference in means).	Transparent methods (Supplementary)
Synthesis of results	14	Describe the methods of handling data and combining results of studies, if done, including measures of consistency (e.g., I^2) for each meta-analysis.	Transparent methods (Supplementary)

Section/topic	#	Checklist item	Reported on page #
Risk of bias across studies	15	Specify any assessment of risk of bias that may affect the cumulative evidence (e.g., publication bias, selective reporting within studies).	Transparent methods (Supplementary)
Additional analyses	16	Describe methods of additional analyses (e.g., sensitivity or subgroup analyses, meta-regression), if done, indicating which were pre-specified.	Transparent methods (Supplementary)
**RESULTS**			
Study selection	17	Give numbers of studies screened, assessed for eligibility, and included in the review, with reasons for exclusions at each stage, ideally with a flow diagram.	5, Suppl Fig. 1
Study characteristics	18	For each study, present characteristics for which data were extracted (e.g., study size, PICOS, follow-up period) and provide the citations.	5, Suppl. Table 1
Risk of bias within studies	19	Present data on risk of bias of each study and, if available, any outcome level assessment (see item 12).	6, Suppl. Table 2
Results of individual studies	20	For all outcomes considered (benefits or harms), present, for each study: (a) simple summary data for each intervention group (b) effect estimates and confidence intervals, ideally with a forest plot.	Figures 1-3 and Supplementary
Synthesis of results	21	Present results of each meta-analysis done, including confidence intervals and measures of consistency.	6-8
Risk of bias across studies	22	Present results of any assessment of risk of bias across studies (see Item 15).	6-8

Additional analysis	23	Give results of additional analyses, if done (e.g., sensitivity or subgroup analyses, meta-regression [see Item 16]).	6-8 and Supplementary
**DISCUSSION**			
Summary of evidence	24	Summarize the main findings including the strength of evidence for each main outcome; consider their relevance to key groups (e.g., healthcare providers, users, and policy makers).	9-13
Limitations	25	Discuss limitations at study and outcome level (e.g., risk of bias), and at review-level (e.g., incomplete retrieval of identified research, reporting bias).	9-13
Conclusions	26	Provide a general interpretation of the results in the context of other evidence, and implications for future research.	9-13
**FUNDING**			
Funding	27	Describe sources of funding for the systematic review and other support (e.g., supply of data); role of funders for the systematic review.	14

From: Moher D, Liberati A, Tetzlaff J, Altman DG, The PRISMA Group (2009). Preferred Reporting Items for Systematic Reviews and Meta-Analyses: The PRISMA Statement. PLoS Med 6(7): e1000097. doi:10.1371/journal.pmed1000097

For more information, visit: **www.prisma-statement.org**.

Strings used for the PubMed search

1- "covid 19"[MeSH Terms] AND (("convalesce"[All Fields] OR "convalesced"[All Fields] OR "convalescence"[MeSH Terms] OR "convalescence"[All Fields] OR "convalescences"[All Fields] OR "convalescent"[All Fields] OR "convalescents"[All Fields] OR "convalescing"[All Fields]) AND ("plasma"[MeSH Terms] OR "plasma"[All Fields] OR "plasmas"[All Fields] OR "plasma s"[All Fields]))
   Translations
   Covid-19[MeSH Terms]: "covid-19"[MeSH Terms]
   convalescent: "convalesce"[All Fields] OR "convalesced"[All Fields] OR "convalescence"[MeSH Terms] OR "convalescence"[All Fields] OR "convalescences"[All Fields] OR "convalescent"[All Fields] OR "convalescents"[All Fields] OR "convalescing"[All Fields]
   plasma: "plasma"[MeSH Terms] OR "plasma"[All Fields] OR "plasmas"[All Fields] OR "plasma's"[All Fields]
   Warnings: SARS-CoV-2 and plasma therapy. Stop word: and
2- "covid 19"[MeSH Terms] AND (("plasma"[MeSH Terms] OR "plasma"[All Fields] OR "plasmas"[All Fields] OR "plasma s"[All Fields]) AND ("therapeutics"[MeSH Terms] OR "therapeutics"[All Fields] OR "therapies"[All Fields] OR "therapy"[MeSH Subheading] OR "therapy"[All Fields] OR "therapy s"[All Fields] OR "therapys"[All Fields]))
   Translations
   Covid-19[MeSH Terms]: "covid-19"[MeSH Terms]
   plasma: "plasma"[MeSH Terms] OR "plasma"[All Fields] OR "plasmas"[All Fields] OR "plasma's"[All Fields]
   therapy: "therapeutics"[MeSH Terms] OR "therapeutics"[All Fields] OR "therapies"[All Fields] OR "therapy"[Subheading] OR "therapy"[All Fields] OR "therapy's"[All Fields] OR "therapys"[All Fields]
   Warnings: SARS-CoV-2 and plasma therapy. Stop word: and
3- ("sars cov 2"[MeSH Terms] OR "sars cov 2"[All Fields] OR "sars cov 2"[All Fields]) AND ("plasma"[MeSH Terms] OR "plasma"[All Fields] OR "plasmas"[All Fields] OR "plasma s"[All Fields]) AND ("therapeutics"[MeSH Terms] OR "therapeutics"[All Fields] OR "therapies"[All Fields] OR "therapy"[MeSH Subheading] OR "therapy"[All Fields] OR "therapy s"[All Fields] OR "therapys"[All Fields])
   Translations
   SARS-CoV-2: "sars-cov-2"[MeSH Terms] OR "sars-cov-2"[All Fields] OR "sars cov 2"[All Fields]
   plasma: "plasma"[MeSH Terms] OR "plasma"[All Fields] OR "plasmas"[All Fields] OR "plasma's"[All Fields]
   therapy: "therapeutics"[MeSH Terms] OR "therapeutics"[All Fields] OR "therapies"[All Fields] OR "therapy"[Subheading] OR "therapy"[All Fields] OR "therapy's"[All Fields] OR "therapys"[All Fields]
   Warnings: SARS-CoV-2 and plasma therapy. Stop word: and
4- ("covid 19"[All Fields] OR "covid 19"[MeSH Terms] OR "covid 19 vaccines"[All Fields] OR "covid 19 vaccines"[MeSH Terms] OR "covid 19 serotherapy"[All Fields] OR "covid 19 serotherapy"[Supplementary Concept] OR "covid 19 nucleic acid testing"[All Fields] OR

"covid 19 nucleic acid testing"[MeSH Terms] OR "covid 19 serological testing"[All Fields] OR "covid 19 serological testing"[MeSH Terms] OR "covid 19 testing"[All Fields] OR "covid 19 testing"[MeSH Terms] OR "sars cov 2"[All Fields] OR "sars cov 2"[MeSH Terms] OR "severe acute respiratory syndrome coronavirus 2"[All Fields] OR "ncov"[All Fields] OR "2019 ncov"[All Fields] OR (("coronavirus"[MeSH Terms] OR "coronavirus"[All Fields] OR "cov"[All Fields]) AND 2019/11/01:3000/12/31[Date - Publication])) AND ("convalesce"[All Fields] OR "convalesced"[All Fields] OR "convalescence"[MeSH Terms] OR "convalescence"[All Fields] OR "convalescences"[All Fields] OR "convalescent"[All Fields] OR "convalescents"[All Fields] OR "convalescing"[All Fields]) AND ("plasma"[MeSH Terms] OR "plasma"[All Fields] OR "plasmas"[All Fields] OR "plasma s"[All Fields]) AND ("mortality"[MeSH Terms] OR "mortality"[All Fields] OR "mortalities"[All Fields] OR "mortality"[MeSH Subheading])

Translations

Covid-19: ("COVID-19" OR "COVID-19"[MeSH Terms] OR "COVID-19 Vaccines" OR "COVID-19 Vaccines"[MeSH Terms] OR "COVID-19 serotherapy" OR "COVID-19 serotherapy"[Supplementary Concept] OR "COVID-19 Nucleic Acid Testing" OR "covid-19 nucleic acid testing"[MeSH Terms] OR "COVID-19 Serological Testing" OR "covid-19 serological testing"[MeSH Terms] OR "COVID-19 Testing" OR "covid-19 testing"[MeSH Terms] OR "SARS-CoV-2" OR "sars-cov-2"[MeSH Terms] OR "Severe Acute Respiratory Syndrome Coronavirus 2" OR "NCOV" OR "2019 NCOV" OR (("coronavirus"[MeSH Terms] OR "coronavirus" OR "COV") AND 2019/11/01[PDAT] : 3000/12/31[PDAT]))

convalescent: "convalesce"[All Fields] OR "convalesced"[All Fields] OR "convalescence"[MeSH Terms] OR "convalescence"[All Fields] OR "convalescences"[All Fields] OR "convalescent"[All Fields] OR "convalescents"[All Fields] OR "convalescing"[All Fields]

plasma: "plasma"[MeSH Terms] OR "plasma"[All Fields] OR "plasmas"[All Fields] OR "plasma's"[All Fields]

mortality: "mortality"[MeSH Terms] OR "mortality"[All Fields] OR "mortalities"[All Fields] OR "mortality"[Subheading]

Warnings: Covid-19 and convalescent plasma and mortality. Stop words: and, and
